# Buckling Sensitivity of Tow-Steered Plates Subjected to Multiscale Defects by High-Order Finite Elements and Polynomial Chaos Expansion

**DOI:** 10.3390/ma14112706

**Published:** 2021-05-21

**Authors:** Alberto Racionero Sanchez-Majano, Alfonso Pagani, Marco Petrolo, Chao Zhang

**Affiliations:** 1 Department of Mechanical and Aerospace Engineering, Politecnico di Torino, 10129 Turin, Italy; alberto.racionero@polito.it (A.R.S.-M.); marco.petrolo@polito.it (M.P.); 2 Joint International Research Laboratory of Impact Dynamics and Its Engineering Applications, Northwestern Polytechnical University, Xi’an 710072, China; chaozhang@nwpu.edu.cn; 3 School of Civil Aviation, Northwestern Polytechnical University, Taicang 215400, China

**Keywords:** sensitivity, multiscale, stochastic fields, Polynomial Chaos, layer-wise models, Unified Formulation

## Abstract

It is well known that fabrication processes inevitably lead to defects in the manufactured components. However, thanks to the new capabilities of the manufacturing procedures that have emerged during the last decades, the number of imperfections has diminished while numerical models can describe the ground truth designs. Even so, a variety of defects has not been studied yet, let alone the coupling among them. This paper aims to characterise the buckling response of Variable Stiffness Composite (VSC) plates subjected to spatially varying fibre volume content as well as fibre misalignments, yielding a multiscale sensitivity analysis. On the one hand, VSCs have been modelled by means of the Carrera Unified Formulation (CUF) and a layer-wise (LW) approach, with which independent stochastic fields can be assigned to each composite layer. On the other hand, microscale analysis has been performed by employing CUF-based Mechanics of Structure Genome (MSG), which was used to build surrogate models that relate the fibre volume fraction and the material elastic properties. Then, stochastic buckling analyses were carried out following a multiscale Monte Carlo analysis to characterise the buckling load distributions statistically. Eventually, it was demonstrated that this multiscale sensitivity approach can be accelerated by an adequate usage of sampling techniques and surrogate models such as Polynomial Chaos Expansion (PCE). Finally, it has been shown that sensitivity is greatly affected by nominal fibre orientation and the multiscale uncertainty features.

## 1. Introduction

Novel fabrication techniques are leading to a reduction in the number of defects present at the mesoscale level of variable stiffness composites (VSCs). One of those procedures is Continuous Tow Shearing (CTS) [1], which permits the steering of the fibres while avoiding waviness and skipping the formation of gaps and/or overlaps among tows. Another arising manufacturing process is the so-called Fused Deposition Modelling (FDM) [2], which is one technique from the vast family of the now named “3D Printing” methodologies. These techniques are promising, yet they are not flaw-exempt. For instance, CTS produces variability in the lamina thickness along the steering direction that stems from the shearing when tows are being placed [3]. Similarly, FDM presents a wide variety of defects. Among them, the most common are voids between layers and surface roughness [4,5]. An extensive review of 3D printing-FDM has been provided by Wickramasinghe et al. [6], in which mechanical properties and defects arising from such procedure are thoroughly depicted.

Many papers have been published where gaps and overlaps due to the Automatic Fibre Placement (AFP) process were accounted for. See, for instance, the works by Blom et al. [7] and Falcò et al. [8] in which a very accurate description of gaps and overlaps was provided. A reduction in terms of computational effort was proposed by Fayazbakhsh et al. [9], employing the so-called Defect Layer Method (DLM). DLM assigns homogenised material properties to the finite elements depending on the presence of gaps or overlaps in the structural discretisation.

The presence of gaps and/or overlaps depends on deterministic parameters such as the turning radius, tow width and the orientation angles of the fibre path and, therefore, their influence can be analysed straightforwardly. However, other sorts of defects might be uncertain in the sense that their location cannot be foreseen beforehand. Moreover, uncertainty is not only induced during the manufacturing of composite components. It can also stem from variability in the elastic properties of the inner constituents, that is, the actual material. Many works have considered the macroscale mechanical properties (see, for instance, the article by Dey et al. [10]), that is, those that are imposed homogeneously to the whole structure regardless of the spatial variation that may exist. Zhou and Gosling [11] studied the uncertainty in the mechanical performance of VSC due to variability in the material properties and in the fibre tow-paths. However, these macroscale properties are due to the mechanical characteristics of the inner constituents, which, in turn, are subjected to uncertainty.

In this context, during the last decades, increasing interest in spatially distributing the uncertainty has appeared. These techniques are known as random or stochastic fields [12] and have already been applied for the study of aerospace structures, as devised in the works by van den Broek et al. [13,14], and Scarth et al. [15]. In the cited articles, material property and geometric variations were induced to investigate how they may affect the free vibration and buckling performance of the structures. Then, in a work by Guimaraes et al. [16], the influence of fibre volume fraction on the flutter instability of wings was analysed. It is worth commenting that in those cited works, different approaches to stochastic field implementation were considered. For instance, in references [13,14,15] the Covariance Matrix Decomposition (CMD) was used, whilst [16] utilised the Karhunen-Loève Expansion (KLE). More information regarding different implementations can be found in the article by Spanos and Zeldin [17].

Similar to the work by Guimaraes et al. [16], other authors included uncertainty at the microscale level. This is relevant since composites are multiscale and hierarchical materials in which the inner constituents influence the mechanical performance of the overall structure and vice versa. One of those works was the research conducted by Naskar et al. [18], in which a comparison between including macromechanical and micromechanical uncertainty was carried out. In this work, they demonstrated that spatially varying properties lead to wider response bounds with the same level of stochasticity, which is explained by the cascading effect of considering uncertainty at the most elementary level of the multiscale. In recent years, Li et al. [19,20] developed a methodology to analyse the different scales of composite materials following a multi-level and multi-site mesh refinement that, as its authors mention, can be used to study the presence of microscopic inclusions and voids.

The presence of such imperfections leads to an uncertain structural response, whose quantification might be aimed. This part of the design process is known as Uncertainty Quantification (UQ) and can be carried out by several techniques, being Monte Carlo analysis one of the most widespread methods. However, Monte Carlo requires the computation of a large number of samples. For that reason, surrogate models are used to accelerate the UQ. Polynomial Chaos Expansion (PCE) is one family of metamodels commonly coupled with KLE-generated random fields. Indeed, the Spectral Stochastic Finite Element Method (SSFEM) [21] has been demonstrated to be a suitable technique for the solution of complex, general problems in probabilistic mechanics. However, this method requires solving extensive systems of equations. For instance, if the deterministic model is of size n×n, and the number of terms considered in the PCE is *N*, then the size of the stochastic system would be N×n×N×n. Other techniques have been proposed to circumvent such numerical issues. For example, Huang et al. [22] employed a collocation scheme to calculate the coefficients of the PCE, which helped to decouple the finite element and stochastic simulations. Therefore, the finite element solver was treated as a black-box model. Apart from structural mechanics, this black-box approach has also been applied to acoustic problems as in the paper by Sharma et al. [23], where they investigated the effect of uncertainties in material and geometric parameters on the acoustic performance of a viscoelastic coating. Indeed, Sharma and Sarkar [24,25] demonstrated that the acoustic radiation could be redirected towards regions of interest by distributing lumped masses.

In this manuscript, the Carrera Unified Formulation (CUF) [26] is utilised for the numerical modelling of VSC plates to exploit its capabilities of creating structural models in which the desired accuracy of the solution is considered as input of the analysis, as demonstrated in [27]. CUF has been employed for a wide variety of problems such as rotor dynamics [28], hygrothermal analysis [29] and incompressible flow analysis [30]. Moreover, CUF has been recently extended to the study of VSC. Vescovini et al. [31,32] employed CUF combined with Ritz methods for free vibration, buckling and post-buckling problems. Then, Demasi et al. [33,34] performed linear static analysis to show the capabilities of CUF against commercial software, demonstrating the reduction in terms of degrees of freedom (DOF). Moreover, Viglietti et al. [35,36] developed one-dimensional models and compared the usage of equivalent single layer (ESL) and layer-wise (LW) theories for the free vibration analysis. Finally, Pagani and Sanchez-Majano [37,38] combined CUF and mesoscale uncertainty to study, respectively, the variability of critical buckling loads and failure indices due to fibre misalignments induced during manufacturing processes. Following the research path established in the previous paragraphs, this manuscript aims to investigate the influence of microscale defects such as the spatially varying fibre volume fraction and the fibre misalignments in the buckling performance of VSC plates and investigate an efficient mathematical model to relate such spatial variation with the macroscale structural response.

The outline of the article is as follows: first, the formulation of layer-wise and component-wise models to describe the macroscale and microscale structure is explained in Section 2. Next, how spatial variation of the micro and mesoscale features are imposed is depicted in Section 3. Then, the uncertainty quantification models are described in Section 4. Afterwards, numerical results are shown in Section 5, and discussed in Section 6. Finally, conclusions are drawn, and comments regarding future developments are made in Section 7.

## 2. Layer- and Component-Wise Unified Finite Elements

In the present study, VSC plates are analysed using one-dimensional (1D) CUF models, which have been extensively used in the structural analysis considering various geometries and materials. Within the CUF framework, the three-dimensional (3D) displacement field can be expressed in terms of an arbitrary expansion of the 1D generalised unknowns that lay along the longitudinal axis, referred to as the *y*-axis hereinafter:

(1) $$u\left(x,y,z\right)=F_{\tau }\left(x,z\right)u_{\tau }\left(y\right)\;\;\tau =1,\ldots ,M.$$

**Figure 1:**
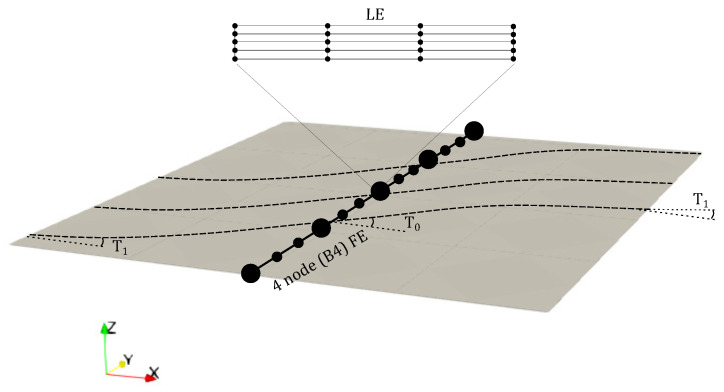
Representation of the finite element (FE) and Lagrange expansion (LE) theories employed over the macroscale structure. FE are used along the longitudinal axis and LE are utilised for the cross-section. $T_{0}$ and $T_{1}$ represent the fibre orientation at the centre of the plate and at the edge, respectively.

Therein, $u_{\tau }\left(y\right)$ represents the vector containing the generalised displacements, $F_{\tau }\left(x,z\right)$ is the expansion function depending on the cross-section coordinates, and *M* is the number of expansion terms. In this manuscript, two expansion functions are utilised, namely the Lagrange expansion (LE) and Hierarchical Legendre expansion (HLE). Such families are explained in upcoming sections. A graphical representation of the spatial discretisation of the macroscale structure is shown in Figure 1.

### 2.1. Finite Element Formulation

In the finite element (FE) method, the generalised displacements $u_{\tau }$ can be expressed in terms of the unknown nodal vector $u_{\tau i}$ and the shape functions $N_{i}\left(y\right)$ as follows:

(2) $$u_{\tau }=N_{i}\left(y\right)u_{\tau i}\;\;i=1,\ldots ,n_{nodes},$$

in which $n_{nodes}$ represents the total number of beam nodes. Lagrange interpolation polynomials are employed as shape functions in this work. For the sake of brevity, these expressions are not reported here, but they can be found in Chapter 8 of the book by Carrera et al. [26]. Then, coupling Equations (1) and (2), one obtains that the generalised 3D displacement field can be expressed as:

(3) $$u\left(x,y,z\right)=F_{\tau }\left(x,z\right)N_{i}\left(y\right)u_{\tau i}\;\;i=1,\ldots ,n_{nodes}\;\tau =1,\ldots ,M.$$

This displacement field can be used along with the principle of virtual displacements (PVD) to derive the governing equations and calculate the stiffness matrix for a linear static problem. According to the PVD:

(4) $$\delta L_{int}=\delta L_{ext},$$

in which $\delta L_{int}$ represents the variation of the internal strain energy

(5) $$\delta L_{int}=\int_{V}\delta \varepsilon^{T}\sigma dV,$$

where ε and σ are the strain and stress tensors in the Voigt notation, respectively; and $\delta L_{ext}$ is the virtual work of the external loading:

(6) $$\delta L_{ext}=F_{s}N_{j}\delta u_{sj}^{T}P,$$

where P denotes the 3×1 vector of the applied load.

Equation (5) can be expanded by using Equation (3), the constitutive relations between stresses and strains and the geometrical relations, yielding the following result:

(7) $$\delta L_{int}=\delta u_{sj}k_{0}^{ij\tau s}u_{\tau i},$$

in which $k_{0}^{ij\tau s}$ is the so-called fundamental nucleus (FN), which is a 3×3 matrix, whose expression is invariant regardless of the structural order and expansion function. The mathematical expression for the FN is:

(8) $$k_{0}^{ij\tau s}=\int_{V}D^{T}\left(F_{\tau }N_{i}\right)\tilde{C}D\left(F_{s}N_{j}\right)dV,$$

where D represents the differential operator containing the geometrical relations between strains and displacements and $\tilde{C}$ is the material stiffness matrix, dependent on the point-wise fibre orientation θ(x,y) and expressed in the global reference frame. A reference on how $\tilde{C}$ can be calculated is found in Reference [38]. The final stiffness matrix $\tilde{k}$ of the structure is assembled by simply looping through the indices *i*, *j*, τ and *s*. Then, the buckling analysis consists in solving the equation:

(9) $$|k_{T}|=0,$$

where $k_{T}$ is the tangent matrix of the structure. The expression for this matrix is derived by means of the PVD:

(10) $$\delta^{2}\left(L_{int}\right)=\int_{V}\delta \left(\delta \varepsilon^{T}\sigma \right)dV=\int_{V}\left[\delta \left(\delta \varepsilon^{T}\right)\sigma +\delta \varepsilon^{T}\delta \sigma \right]dV.$$

After applying the expressions from Equation (3), the constitutive law and the geometrical relations, the previous equation adopts the following form:

(11) $$\delta^{2}\left(L_{int}\right)=\delta u_{sj}^{T}k_{T}^{ij\tau s}\delta u_{\tau i}.$$

This equation can be written for the case of linearised buckling problem as:

(12) $$\delta^{2}\left(L_{int}\right)\approx \delta u_{sj}^{T}\left(k_{0}^{ij\tau s}+k_{\sigma }^{ij\tau s}\right)\delta u_{\tau i},$$

where the tangent stiffness matrix has been expressed as $k_{T}^{ij\tau s}=k_{0}^{ij\tau s}+k_{\sigma }^{ij\tau s}$. On one side, $k_{0}^{ij\tau s}$ refers to the linear stiffness matrix in terms of FN. On the other side, $k_{\sigma }^{ij\tau s}$ represents the FN of the geometric stiffness matrix, which strictly depends on the internal linear stress state of the structure. This stress state will be dependent on the accuracy of the model. The equations that allow the calculation of the tangent matrix are not reported in the manuscript for brevity reasons but can be found in [39]. Finally, since the linear hypothesis holds, $k_{\sigma }$ is supposed to be proportional to $\lambda_{cr}$, which is the solution to the eigenvalue problem and is proportional to the applied load in the case of linearised buckling. Thus, Equation (9) can be rewritten as follows:

(13) $$|k_{0}+\lambda_{cr}k_{\sigma }|=0,$$

in order to calculate $\lambda_{cr}$. Note that $k_{0}$ and $k_{\sigma }$ denote the global assembled finite element arrays.

### 2.2. Cross-Sectional Expansions for Multilayered and Multicomponent Structures

The structural theory depends on the order of the chosen Lagrange polynomial: four-node bilinear L4, nine-node quadratic L9 and cubic sixteen-node sixth-order L16. For instance, the interpolation functions of L4 expansion are defined as:

(14) $$F_{\tau }=\frac{1}{4}\left(1-r_{\tau }r\right)\left(1-s_{\tau }s\right)\;\;\tau =1,2,3,4,$$

where r,s∈[−1,1]×[−1,1], and $r_{\tau }$ and $s_{\tau }$ represent the location of the polynomials’ roots. Thus, the kinematic displacement field of a single L4 beam is:

(15) $$\begin{array}{c} u_{x}\left(x,y,z\right)=F_{1}\left(x,z\right)u_{1x}\left(y\right)+F_{2}\left(x,z\right)u_{2x}\left(y\right)+F_{3}\left(x,z\right)u_{3x}\left(y\right)+F_{4}\left(x,z\right)u_{4x}\left(y\right)\\ u_{y}\left(x,y,z\right)=F_{1}\left(x,z\right)u_{1y}\left(y\right)+F_{2}\left(x,z\right)u_{2y}\left(y\right)+F_{3}\left(x,z\right)u_{3y}\left(y\right)+F_{4}\left(x,z\right)u_{4y}\left(y\right)\\ u_{z}\left(x,y,z\right)=F_{1}\left(x,z\right)u_{1z}\left(y\right)+F_{2}\left(x,z\right)u_{2z}\left(y\right)+F_{3}\left(x,z\right)u_{3z}\left(y\right)+F_{4}\left(x,z\right)u_{4z}\left(y\right), \end{array}$$

where $u_{1x},u_{1y},u_{1z},\ldots ,u_{4x},u_{4y},u_{4z}$ are the generalised displacements.

On the other hand, HLE consists of a set of two-dimensional (2D) Legendre polynomials that act as expansion functions of the cross-section coordinates. This set of interpolation functions was derived by Szabò and Babuska [40] for the *p*-version of the finite element method for the 1D interpolation functions and exhibit some interesting properties for the generation of interpolation functions. They constitute an orthogonal basis and form a fully hierarchical set. The 1D set was later extended to quadrilateral domains by Pagani et al. [41]. Depending on where these polynomials vanish, they are divided into the following categories:

- Nodal modes: they are analogous to the Lagrange linear interpolation polynomials on the four vertex nodes of the quadrilateral domain. They vanish in all vertices, but one, and their expressions are:
  (16) $$F_{\tau }\left(r,s\right)=\frac{1}{4}\left(1-r_{i}r\right)\left(1-s_{i}s\right)\;\mathrm{for}\;i=1,2,3,4.$$
- Side modes: they are defined for p≥2. They vanish for all edges, but one, and are defined in the [−1,1] × [−1,1] domain as:
  (17) $$\begin{array}{cc} & F_{\tau }\left(r,s\right)=\frac{1}{2}\left(1-s\right)\phi_{p}\left(r\right)\;\mathrm{for}\;i=5,9,13,18 \end{array}$$
  (18) $$\begin{array}{cc} & F_{\tau }\left(r,s\right)=\frac{1}{2}\left(1+r\right)\phi_{p}\left(s\right)\;\mathrm{for}\;i=6,10,14,19 \end{array}$$
  (19) $$\begin{array}{cc} & F_{\tau }\left(r,s\right)=\frac{1}{2}\left(1+s\right)\phi_{p}\left(r\right)\;\mathrm{for}\;i=7,11,15,20 \end{array}$$
  (20) $$\begin{array}{cc} & F_{\tau }\left(r,s\right)=\frac{1}{2}\left(1-r\right)\phi_{p}\left(s\right)\;\mathrm{for}\;i=8,14,16,21, \end{array}$$
  where $\phi_{p}$ corresponds to the 1D internal Legendre-type modes, defined in [40].
- Internal modes: they are built by multiplying 1D internal modes. They are considered when the polynomial has a degree p≥4, and vanish at all the edges of the domain. For instance, the set of sixth-order polynomials comprises three internal expansions are:
  (21) $$\begin{array}{cc} & F_{28}\left(r,s\right)=\phi_{4}\left(r\right)\phi_{2}\left(s\right) \end{array}$$
  (22) $$\begin{array}{cc} & F_{29}\left(r,s\right)=\phi_{3}\left(r\right)\phi_{3}\left(s\right) \end{array}$$
  (23) $$\begin{array}{cc} & F_{30}\left(r,s\right)=\phi_{2}\left(r\right)\phi_{4}\left(s\right). \end{array}$$

**Figure 2:**
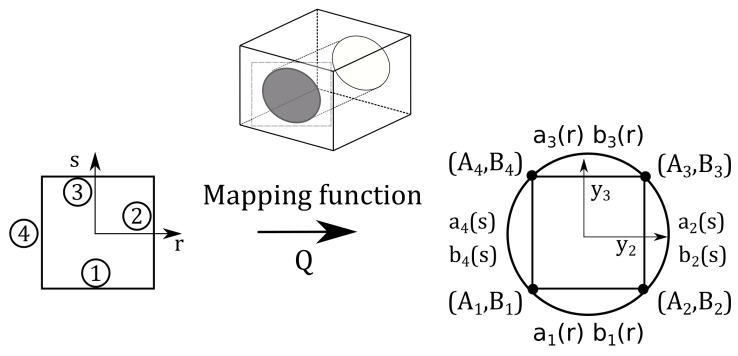
Mapping of the fibre section of the RUC by means of the blending function method. Reprinted from Reference [42], with permission from Elsevier.

HLE theories can be used to obtain a coarse discretisation over large quadrilateral domains. However, when dealing with curved geometries, standard isoparametric elements represent the boundaries employing the same interpolation functions, thus introducing a numerical error while computing the stiffness matrix due to the inability to exactly represent the curved boundaries. In the case of large curved domains, this error can be sufficiently large to consider the usage of non-isoparametric techniques to represent such boundaries. This is of utmost importance when, over cross-sectional domains, there exist diverse phases with different material properties as illustrated in Figure 2.

Several mapping techniques exist, such as the so-called first-order and second-order mapping. Nevertheless, if an exact representation is pursued, the blending function method (BFM) developed by Gordon and Hall [43] is recommended. BFM permits to describe the exact geometry of an arbitrary component in the cross-sectional coordinates $y_{2},y_{3}$ by means of the mapping functions as:

(24) $$\begin{array}{c} y_{2}=Q_{a}\left(r,s\right)=\frac{1}{2}\left(1-s\right)a_{1}\left(r\right)+\frac{1}{2}\left(1+r\right)a_{2}\left(s\right)+\frac{1}{2}\left(1+s\right)a_{3}\left(r\right)+\frac{1}{2}\left(1-r\right)a_{4}\left(s\right)-F_{\tau }\left(r,s\right)r_{\tau } \end{array}$$

(25) $$\begin{array}{c} y_{3}=Q_{b}\left(r,s\right)=\frac{1}{2}\left(1-s\right)b_{1}\left(r\right)+\frac{1}{2}\left(1+r\right)b_{2}\left(s\right)+\frac{1}{2}\left(1+s\right)b_{3}\left(r\right)+\frac{1}{2}\left(1-r\right)ab_{4}\left(s\right)-F_{\tau }\left(r,s\right)s_{\tau }, \end{array}$$

where τ=1,…,4 and $a_{\tau }$ and $b_{\tau }$ are the parametric curves of the edges. For further insights into these mapping techniques, the reader is invited to read [41], where these methodologies are properly explained.

The assembly of multilayered and multicomponent structures’ stiffness arrays is discussed in the following. For instance, LW models allow the consideration of the generalised displacements of each individual layer independently. Then, compatibility conditions are imposed at the interfaces of two consecutive plies by considering that:

(26) $$u_{top}^{k}=u_{bottom}^{k+1},$$

in which *k* represents the *k*-th layer of the laminate. This model was initially introduced employing HLE by Pagani et al. [41] for the analysis of classical laminates and thin-walled structures and, more recently, LE was used for the study of VSC in the works by Viglietti et al. [35,36] and Pagani and Sanchez-Majano [37,38].

**Figure 3:**
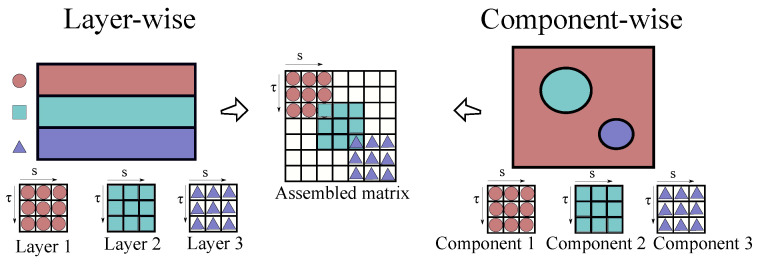
Layer-wise and component-wise assembly procedures.

Based on this approach, and taking advantage of the CUF capacities, the LW modelling can be extended straightforwardly to any component on the cross-section with no loss of generalisation. Indeed, by extending the meaning of the index *k* from the layer to a generic component of the cross-section, one can generate independent kinematics for the matrix, the fibre or any other component and then impose the compatibility of displacements at the interfaces. Thus, the assembly of the stiffness matrix of a component-wise (CW) model remains formally the same as that of LW approaches. Both assembly procedures are illustrated in Figure 3.

### 2.3. Micromechanical Modelling

**Figure 4:**
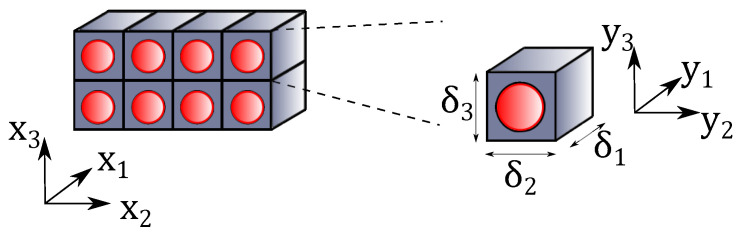
Representation of a periodic heterogeneous material and its RUC along with the global and local coordinate reference frames.

Composite structures can be considered as a whole ensemble of microstructures periodically distributed over the structure’s volume. In this context, the Representative Unit Cell (RUC) constitutes the essential building block that contains the necessary information to identify the material properties. This is represented in Figure 4, where a zoom into the heterogeneous material shows the RUC. The macroscopic properties can be defined in the global reference system $x=x_{1},x_{2},x_{3}$, whereas $y=y_{1},y_{2},y_{3}$ denotes the local reference frame of the RUC. Micromechanical analyses can be two-way: (i) in a first instance, they can be used to calculate the effective properties of the heterogeneous material represented by the RUC as input of the equivalent homogeneous material properties in higher scale analyses; (ii) retrieve the displacements, strains and stresses fields over the RUC from the outputs of the macroscale structural analysis at certain points of interest.

Micromechanical analyses assume that the RUC is much smaller than the macroscopic structure, such that y=x/δ, where δ is a scaling factor that characterises the dimension of the RUC. In micromechanics, the material properties provided by the RUC analysis at the microscale do not depend on the macroscale structural problem. That is, they are intrinsic properties of the material chosen for the structural analysis. Additionally, the average value of the local solutions over the RUC volume is equal to the global solution of the macroscopic problem. That is, for a generic field ϕ(x,y):

(27) $$\frac{1}{V}\int_{V}\phi \left(x,y\right)dV=\bar{\phi }\left(x\right),$$

where *V* is the volume of the RUC, ϕ(x,y) is the local field, dependent of the global and local coordinates (x and y, respectively) and $\bar{\phi }$ is the averaged field which only depends on the global coordinates. Generally, periodic boundary conditions are applied to guarantee the compatibility of deformations with respect to the adjacent RUCs. This periodicity can be written as:

(28) $$\begin{array}{c} u_{i}\left(x_{1},x_{2},x_{3};\delta_{1}/2,y_{2},y_{3}\right)=u_{i}\left(x_{1}+\delta_{1},x_{2},x_{3};-\delta_{1}/2,y_{2},y_{3}\right)\\ u_{i}\left(x_{1},x_{2},x_{3};y_{1},\delta_{2}/2,y_{3}\right)=u_{i}\left(x_{1},x_{2}+\delta_{2},x_{3};y_{1},-\delta_{2}/2,y_{3}\right)\\ u_{i}\left(x_{1},x_{2},x_{3};y_{1},y_{2},\delta_{3}/2\right)=u_{i}\left(x_{1},x_{2},x_{3}+\delta_{3};y_{1},y_{2},-\delta_{3}/2\right). \end{array}$$

In this work, the Variational Asymptotic Method (VAM) and the Mechanics of Structure Genome (MSG), initially derived in [44,45], are coupled with CUF to obtain the homogenised material properties of the RUC. MSG states that such properties of an RUC can be obtained by minimising the difference between the strain energies of the heterogeneous structure and the equivalent homogeneous material. This difference is expressed as the following functional:

(29) $$\Pi =\frac{1}{V}\int_{V}\frac{1}{2}C_{ijkl}\varepsilon_{ij}\varepsilon_{kl}dV-\frac{1}{2}C_{ijkl}^{*}{\bar{\varepsilon }}_{ij}{\bar{\varepsilon }}_{kl},$$

where the first term is the strain energy of the heterogeneous material represented by the RUC, whilst the second corresponds to the strain energy of the homogeneous one. $C_{ijkl}$ represents the fourth-order elastic tensor, and $\varepsilon_{ij}$ is the second-order strain tensor. Similarly, $C_{ijkl}^{*}$ and ${\bar{\varepsilon }}_{ij}$ are their equivalents of the homogeneous material, respectively. In micromechanics, the local displacements $u_{i}\left(x;y\right)$ can be written in terms of the global displacements ${\bar{u}}_{i}\left(x\right)$ and a fluctuation $\chi_{i}$, which is scaled down by δ, as:

(30) $$u_{i}\left(x;y\right)={\bar{u}}_{i}\left(x\right)+\delta \chi_{i}\left(x;y\right).$$

Then, because of the different coordinate systems involved in a multiscale analysis, the derivative of a field ϕ(x;y) can be computed as:

(31) $$\frac{\partial \phi }{\partial x_{j}}+\frac{1}{\delta }\frac{\partial \phi }{\partial y_{j}}.$$

Thus, applying Equation (31) to Equation (30), and neglecting small terms according to VAM, the strains can be expressed as:

(32) $$\varepsilon_{ij}\left(x;y\right)={\bar{\varepsilon }}_{ij}\left(x\right)+\chi_{i,j}\left(x;y\right),$$

where

(33) $${\bar{\varepsilon }}_{ij}\left(x\right)=\frac{1}{2}\left(\frac{\partial {\bar{u}}_{i}\left(x\right)}{\partial x_{j}}+\frac{\partial {\bar{u}}_{j}\left(x\right)}{\partial x_{i}}\right)$$

and

(34) $$\chi_{i,j}\left(x,y\right)=\frac{1}{2}\left(\frac{\partial \chi_{i}\left(x;y\right)}{\partial x_{j}}+\frac{\partial \chi_{j}\left(x;y\right)}{\partial x_{i}}\right).$$

Then, using Equation (27), the following can be written:

(35) $${\bar{u}}_{i}=\langle u_{i}\rangle \;\;\;{\bar{\varepsilon }}_{ij}=\langle \varepsilon_{ij},\rangle$$

which yields the following constraints to the fluctuation unknowns:

(36) $$\langle \chi_{i}\rangle =0\;\;\;\langle \chi_{i,j}\rangle =0.$$

Finally, making use of the displacement and strain expressions from Equations (30) and (32), respectively, and considering the second term from Equation (29) as a constant, the fluctuation unknowns $\chi_{i}$ can be obtained by minimising the functional:

(37) $$\Pi =\frac{1}{V}\int_{V}\frac{1}{2}C_{ijkl}\left({\bar{\varepsilon }}_{ij}+\chi_{i,j}\right)\left({\bar{\varepsilon }}_{kl}+\chi_{k,l}\right)dV.$$

**Figure 5:**
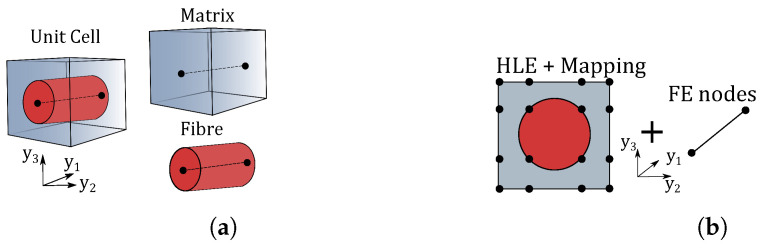
(**a**) Component-wise modelling of composite microstructure with different phases. (**b**) Kinematics used to define the RUC cross-section.

In the CUF micromechanics framework, the RUC is modelled by means of 1D beam elements using the CW approach. Figure 5a shows a composite microstructure with two different constituents and the CW idealisation of it with individual components modelled separately. Figure 5b represents the assembled cross-section with HLE elements along the beam axis for the RUC. The cross-section lies in the $y_{2}-y_{3}$ plane and extends along the longitudinal direction $y_{1}$. The coarse mesh employed for the discretisation of the cross-section is due to the BFM coupling with fourth-order HLE, while for the beam axis, a two-node beam element is used. The geometry of the model is fixed at the beginning of the analysis, and the accuracy of the micromechanical analysis is tuned through the polynomial order of the theory of structure. Readers are referred to the original work by de Miguel et al. [42], where a detailed derivation, in the CUF framework, of the explained micromechanical problem is obtained. Therein, Equation (37) is expressed in terms of FN for the RUC problem.

## 3. Stochastic Fields Using KLE

**Figure 6:**
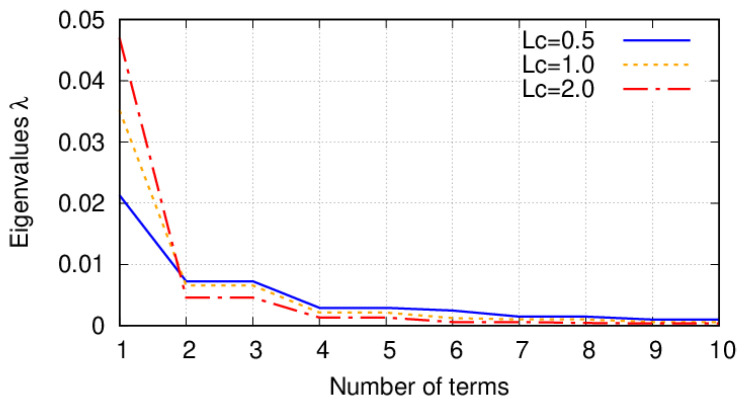
Eigenvalues of the Fredholm integral for different correlation lengths.

**Figure 7:**
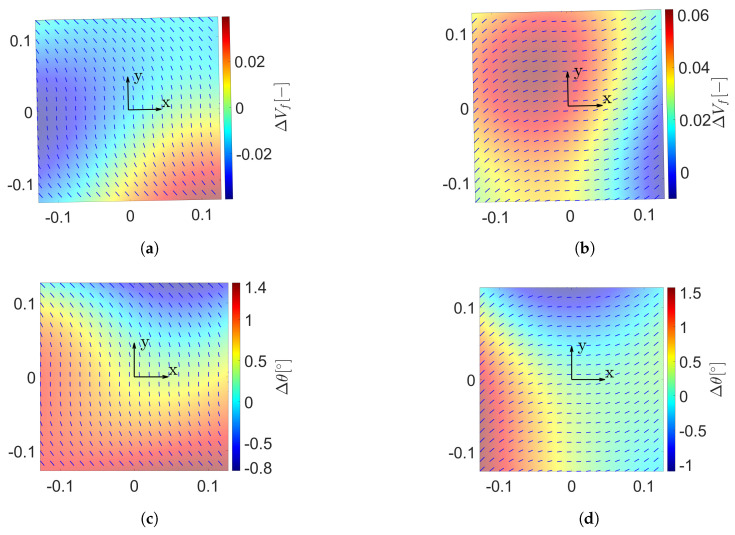
(**a**) Fibre volume fraction ($\Delta V_{f}$) random field over a [90+<0,45>]. (**b**) $\Delta V_{f}$ random field over a [0+<0,45>] ply. (**c**) Misalignment field over a [90+<0,45>] ply. (**d**) Misalignment field over a [0+<0,45>] ply. These random fields are generated by means of the Karhunen-Loève expansion. The stochastic fields represent the fibre volume fraction and has a mean value ${\tilde{V}}_{f}=0.60$ and a standard deviation $\sigma_{V_{f}}=0.05$ and fibre misalignments of null mean and standard deviation $\sigma_{\theta }=1.5^{\circ }$.

In this work, both a spatial variation of the fibre volume fraction at the microscale level and fibre misalignments at the layer level is imposed. This is made by means of a two-dimensional stationary stochastic field dependent on the in-plane coordinates, which, in a general notation, can be expressed as follows:

(38) $$H^{k}\left(x,y;\alpha \right)={\tilde{H}}^{k}+\Delta H^{k}\left(x,y;\alpha \right),$$

in which α represents the random nature of the stochastic distributions and the superscript *k* refers to the *k*-th layer of the laminate; ${\tilde{H}}^{k}$ is the mean value of the stochastic field and $\Delta H^{k}\left(x,y;\alpha \right)$ denotes the Gaussian variation of the random field about its mean for layer *k*. $H^{k}\left(x,y;\alpha \right)$ can represent the fibre volume fraction $V_{f}^{k}$ and the misalignment $\Theta^{k}$. Note that misalignments affect the nominal fibre path θ(x,y), hence $\Theta^{k}=\theta^{k}\left(x,y\right)+\Delta \Theta^{k}\left(x,y;\alpha \right)$. In a generalised manner, the random fluctuation can be expressed in terms of an infinite series expansion referred to as Karhunen-Loève expansion (KLE):

(39) $$\Delta H^{k}\left(x,y;\alpha \right)=\sum_{i=1}^{\infty }\xi_{i}\left(\alpha \right)\sqrt{\lambda_{i}}\varphi_{i}\left(x,y\right),$$

where $\xi_{i}\left(\alpha \right)$ are standard uncorrelated random variables and $\lambda_{i}$ and $\varphi_{i}\left(x,y\right)$ are the eigenvalues and eigenfunctions of the autocovariance kernel from solving the homogeneous Fredholm integral equation of the second kind:

(40) $$\begin{array}{c} \int C\left(x,x^{\prime }\right)\varphi_{i}\left(x^{\prime }\right)dx^{\prime }=\lambda_{i}\varphi_{i}\left(x\right) \end{array}$$

in which $C(x,x^{\prime })$ is the autocovariance kernel of the stochastic field. As stated in the book by Ghanem and Spanos [21], there exist analytical solutions to Equation (40) when certain families of covariance kernel are considered. One of them is the exponential function, which is considered in this work and is expressed as:

(41) $$C\left(x,x^{\prime },y,y^{\prime }\right)=\sigma_{H^{k}}^{2}e^{-|x-x^{\prime }|/l_{x}-\left|y-y^{\prime }\right|/l_{y}},$$

in which $l_{x}$ and $l_{y}$ are the correlation lengths in *x* and *y* direction, respectively. Note that when $l_{x}=l_{y}$, the stochastic field is called isotropic. Then, depending on the value of the correlation lengths, the amount of negligible terms of the truncated KLE varies. This can be appreciated in Figure 6 where the first eigenvalues have higher values when a larger correlation length is considered. Therefore, the higher the correlation length is, the fewer terms could be considered in the KLE. Nevertheless, the value of the correlation length $l_{c}$ is based on the experience of both the engineer and the spatially varying property that is considered. In this work, $l_{x}=l_{y}$ and equal to the plate side length for both stochastic fields. Mean values and standard deviations have been selected according to References [16,46] for fibre volume fraction and fibre waviness, respectively. Examples of these two stochastic fields over VSC plies are illustrated in Figure 7, in which *x* and *y* represent the in-plane coordinates of the plate.

Finally, the closed-form solutions to the stated problem can be found in the aforementioned book [21]. However, numerical approaches such as Nyström and Galerkin finite element method can be found in the paper by Betz et al. [47]. For instance, Galerkin FE was utilised to generate 3D stochastic fields in the paper by Choi et al. [48].

## 4. Polynomial Chaos Expansion

Commonly, uncertainty analyses regarding certain parameters of interest are carried out by means of Monte Carlo analysis due to their relative easiness. Indeed, it consists of computing several ($10^{3}$–$10^{6}$) samples of a deterministic problem in which those parameters are tweaked between each run. Afterwards, statistical moments can be computed. Unfortunately, due to the expensive computational models that current numerical simulations require, performing this sort of analysis is time-consuming. Therefore, advanced techniques such as Polynomial chaos expansion (PCE) are used for such purposes.

In the present study, uncertainty quantification of the critical buckling loads ($F_{cr}$) is carried out by means of PCE. PCE can be used as a response surface metamodel that represents the stochastic output as a multivariate polynomial function of a set of convenient random variables. PCE can be generally expressed as:

(42) $$F_{cr}\left(\xi_{1},\xi_{2},\ldots ,\xi_{r}\right)=a_{0}\Gamma_{0}+\sum_{i=1}^{\infty }a_{1i}\Gamma_{1}\left(\xi_{i1}\left(\alpha \right)\right)+\sum_{i=1}^{\infty }\sum_{j=1}^{i}a_{i1i2}\Gamma_{2}\left(\xi_{i1}\left(\alpha \right)\xi_{i2}\left(\alpha \right)\right)+\ldots ,$$

where $\xi_{i1}\left(\alpha \right)$ is a set of independent standard Gaussian variables and $\Gamma_{p}\left(\xi_{i1}\left(\alpha \right),\ldots ,\xi_{ip}\left(\alpha \right)\right)$ is a set of multivariate Hermite polynomials of order *p*; $a_{i1},\ldots ,a_{ip}$ are deterministic coefficients and α represents the random nature of the quantities involved. Equation (42) can be rearranged and expressed as:

(43) $$F_{cr}\left(\xi_{1},\xi_{2},\ldots ,\xi_{n}\right)=\sum_{i=0}^{r}\beta_{i}\psi_{i}\left(\xi_{i}\left(\alpha \right)\right),$$

in which $\beta_{i}$ and $\psi_{i}\left(\xi_{i}\left(\alpha \right)\right)$ are equivalent to $a_{i1},\ldots ,a_{ip}$ and $\Gamma_{p}\left(\xi_{i1}\left(\alpha \right),\ldots ,\xi_{ip}\left(\alpha \right)\right)$, respectively. Note that, depending on the nature of the uncertainty quantities involved, that is: Gaussian, uniform, beta distribution, and so forth, the polynomial basis varies as depicted in [49]. Finally, the number of terms involved in a PCE up to order *p* are calculated by the following expression:

(44) $$N=\frac{(r+p)!}{r!p!},$$

where *r* is the number of variables involved, and *p* is the polynomial degree. Additionally, due to the orthonormality of the polynomial basis, the first two statistical moments of the PCE are encoded in its coefficients. Therefore, the mean value ${\tilde{F}}_{cr}$ and the variance $\sigma_{F_{cr}}^{2}$ can be calculated using the following expressions:

(45) $$\begin{array}{c} {\tilde{F}}_{cr}=\beta_{0}\\ \sigma_{F_{cr}}^{2}=\sum_{i=1}^{r}\beta_{i}^{2}. \end{array}$$

**Figure 8:**
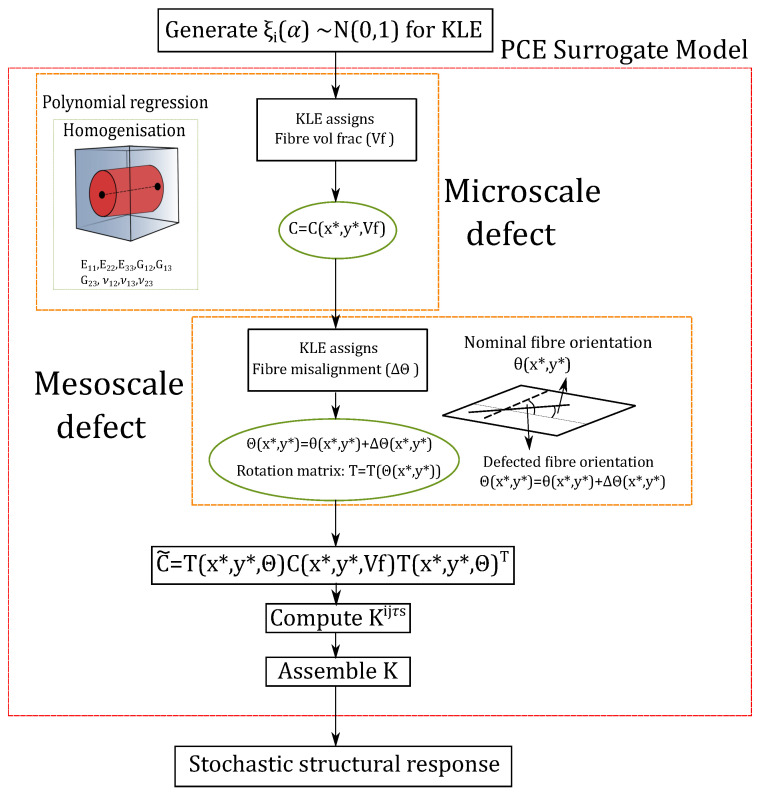
Flow-chart of the stochastic structural analysis performed.

In this study, the PCE independent variables $\xi_{i}\left(\alpha \right)$ correspond to the standard Gaussian terms considered in the KLE (recall Equation (39)), and $F_{cr}$ denote the critical buckling loads whose regression is intended. This uncertainty quantification procedure is illustrated in Figure 8, where the dashed box contains the FE model, which aims to be substituted by the PCE surrogate model. In this manner, the PCE is used as a non-intrusive model. Further details on how the uncertainty is propagated throughout the FE solver is available in Section 4.1. Similarly to the critical buckling loads, other quantities of interest, such as stresses, could be computed by means of PCE.

### 4.1. Multiscale Uncertainty Quantification

**Figure 9:**
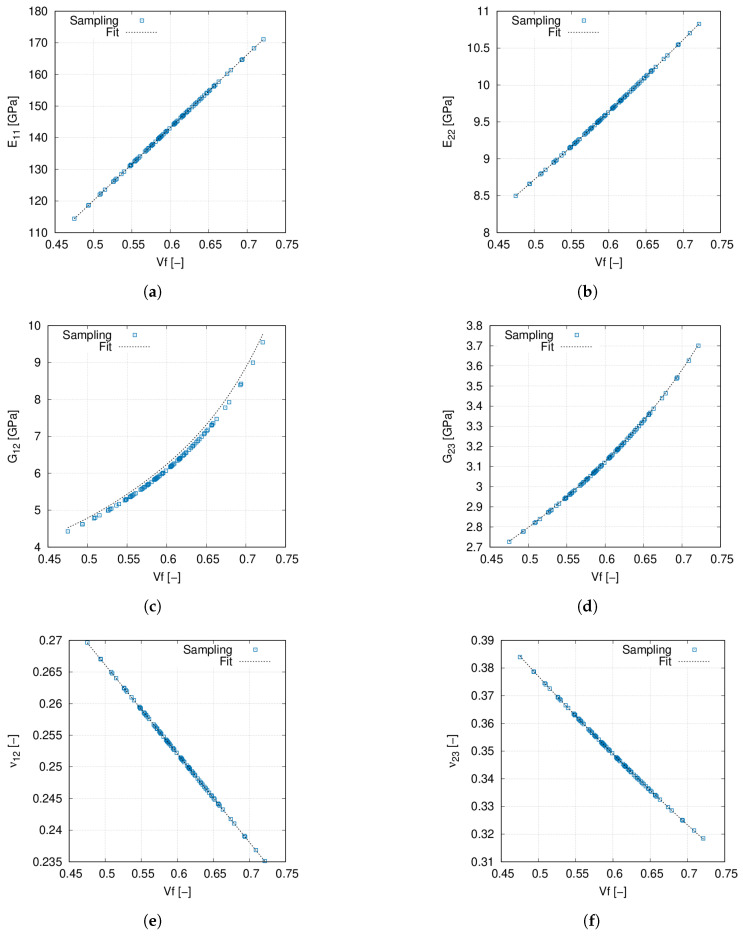
(**a**) $E_{11}$ vs. $V_{f}$, (**b**) $E_{22}$ vs. $V_{f}$, (**c**) $G_{12}$ vs. $V_{f}$, (**d**) $G_{23}$ vs. $V_{f}$, (**e**) $\nu_{12}$ vs. $V_{f}$, (**f**) $\nu_{23}$ vs. $V_{f}$. Sampling results and data fit of the homogenised material properties. Fibre volume fraction is treated as a Gaussian random variable with mean value $V_{f}=0.60$ and standard deviation $\sigma_{V_{f}}=0.05$.

The flow-chart of this multiscale procedure is depicted in Figure 8 and is explained herein. First of all, $r=4n_{\mathrm{def}}n$$\xi_{i}\left(\alpha \right)$ terms for the KLE are generated by means of Latin Hypercube Sampling (LHS) for each analysis run. The reason for generating $4n_{\mathrm{def}}n$ is that the structure that is analysed has four layers and *n* terms for $n_{\mathrm{def}}$ defects are considered in the KLE of each ply’s stochastic fields. Once the structural analysis begins, a fibre volume fraction and fibre misalignment field are obtained with the KLE and are assigned to each integration point. First, an homogenisation of the material properties is carried out by means of a polynomial regression, whose curves are shown in Figure 9. Then, these material properties are used to calculate the coefficients of the material stiffness matrix (*C*), which is then rotated into the structural reference frame taking into account the fibre misalignment ΔΘ to obtain $\tilde{C}$. Afterwards, the FN is computed for each FE, and the global stiffness matrix is assembled. Once the equilibrium state is calculated, the internal stress state is employed to calculate the geometrical stiffness matrix from Equation (13). Finally, the stochastic buckling response is retrieved.

Note that the curves in Figure 9 have been obtained by randomly sampling $10^{2}$ values of $V_{f}$, which is considered as a Gaussian random variable of mean value equal to 0.60 and a standard deviation equal to 0.05. Each sample is used as input of the homogenisation problem, and the homogenised elastic properties are retrieved by means of the methodology proposed in Section 2.3. After sampling, regression curves are obtained by polynomial fit. From these curves, it can be appreciated that larger fibre volume fractions provide higher values of the Young, transverse and shear moduli. Conversely, Poisson ratios decrease with increasing values of $V_{f}$.

As the reader can see, this is a cumbersome procedure in which several numerical techniques are employed and requires high computational cost when larger structures are involved. Therefore, it is convenient to build a surrogate model that accounts for the macroscale behaviour of the structure. As it was mentioned priorly, in this work, PCE is used for such a purpose. In this case, the input of the surrogate model are the $4n_{\mathrm{def}}n$$\xi_{i}\left(\alpha \right)$ coefficients used to build the layers’ random fields and the output are the different buckling loads, where n=10 are the number of terms considered in the KLE per random field. The fact of considering $4n_{\mathrm{def}}n$ terms to build the PCE surrogate is explained in the report by Sudret and Der-Kiureghian [12] in which the inclusion of multiple random fields is discussed. The addition of $n_{\mathrm{def}}$ defects increases the amount of $\xi_{i}\left(\alpha \right)$ coefficients, and thus the size of the PCE basis. This fact may lead to the so-called curse of dimensionality, hence requiring a large number of samples to characterise the buckling load variability thoroughly.

## 5. Numerical Results

**Figure 10:**
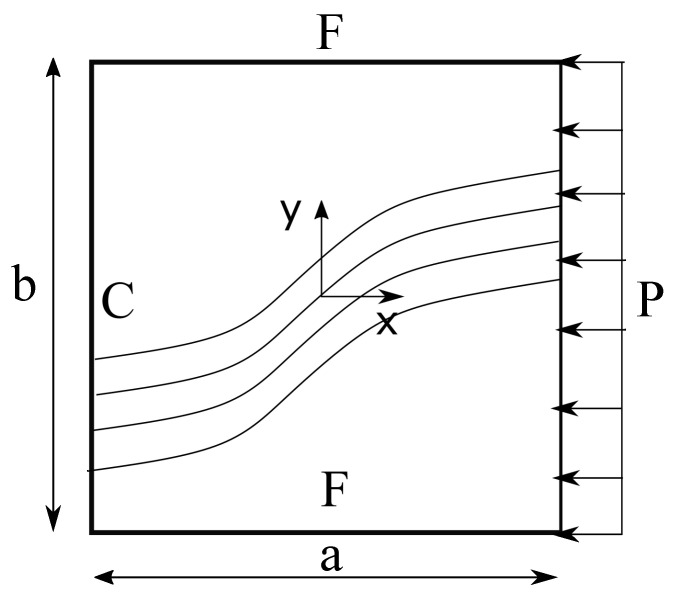
Boundary conditions of the laminated plates. C and F stand for clamped and free edges respectively. Pressure P is exerted uniformly on the laminate yz plane.

This section presents a series of numerical assessments on the verification of the proposed in-house-developed 1D CUF-based FE models used to solve the linearised buckling problem of VSC plates. In these assessments, a four-layer balanced and symmetric variable stiffness composite plate is analysed. The structure is clamped on one edge, while, on the opposite side, a pressure P=7.75 kPa is applied, and the remaining edges are free (see Figure 10), which is equivalent to apply a unitary point-load. The plate dimensions are listed in Table 1 and the material properties of the fibre and matrix are shown in Table 2, along with the homogenised material properties using a nominal fibre volume fraction $V_{f}=0.60$. Recall that these homogenised material properties are the outcome of a micromechanical analysis, in which the RUC is composed of a two-node beam FE and a fourth-order HLE using BFM for the cross-section. Two fibre orientations are considered in this study, namely:

- Case 1: $\theta ={\left[0\pm <45,0>\right]}_{s}$
- Case 2: $\theta ={\left[90\pm <0,45>\right]}_{s}$,

following the notation for linearly varying fibre orientation introduced by Gürdal and Olmedo in [50] as $\theta =[\phi <T_{0},T_{1}>]$. ϕ represents the rotation of the fibre path with respect to the *x*-axis and $T_{0}$ and $T_{1}$ are the orientations at the centre and at the edge of the plate as shown in Figure 1.

### 5.1. Preliminary Assessments on Pristine Plates

The first numerical assessment is the verification of the proposed CUF FE model against the commercial software Abaqus. Initially, a mesh convergence analysis is carried out. In these results, the fibre volume fraction is fixed to $V_{f}=0.60$, and the fibre orientation is not subjected to any misalignments. An equal number of beam elements and cross-section subdomains are used to discretise the *y* and *x* axes, while a single cross-sectional element is used per ply. Quadratic (B3/L9) and cubic (B4/L16) elements are considered for both *y* and *x* directions, thus yielding to the nomenclature *D* B*Y*-*E* L*X*, where *D* and *E* denote the amount of FE and cross-sectional subdomains along the mentioned directions, and *Y* and *X* represent the order of the elements, respectively.

The outcomes of the mesh convergence analysis are shown in Table 3 and Table 4, along with the results of a similar study conducted using Abaqus, which utilises a planar shell model composed of S4R elements.

In addition to the previous results, the computational time has been taken into account since, in the following assessments, time plays a significant role. Table 5 shows the relative computational time, with respect to the computing time of the 4B4-4L16 mesh, which has been chosen to perform the following sensitivity analyses. Each of the simulations has been carried out by an i7-10510U CPU 1.80 GHz.

**Figure 11:**
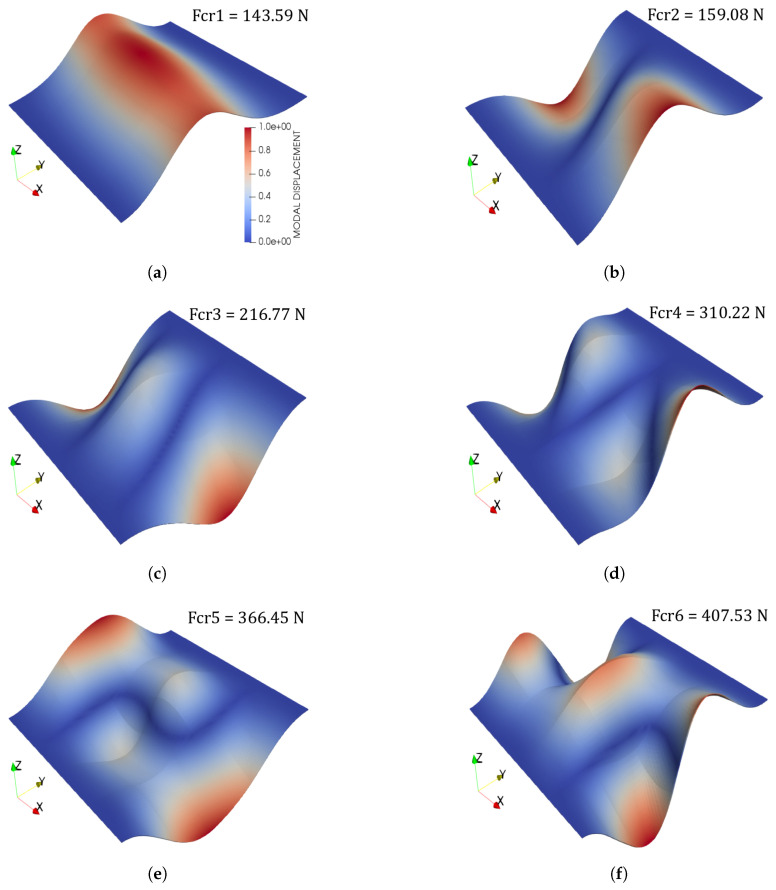
(**a**) First mode, (**b**) Second mode, (**c**) Third mode, (**d**) Fourth mode, (**e**) Fifth mode, (**f**) Sixth mode. Case 1 pristine buckling modes. A uniform fibre volume $V_{f}=0.60$ is considered and no fibre waviness. The colour bar in (**a**) applies for all figures in this panel.

**Figure 12:**
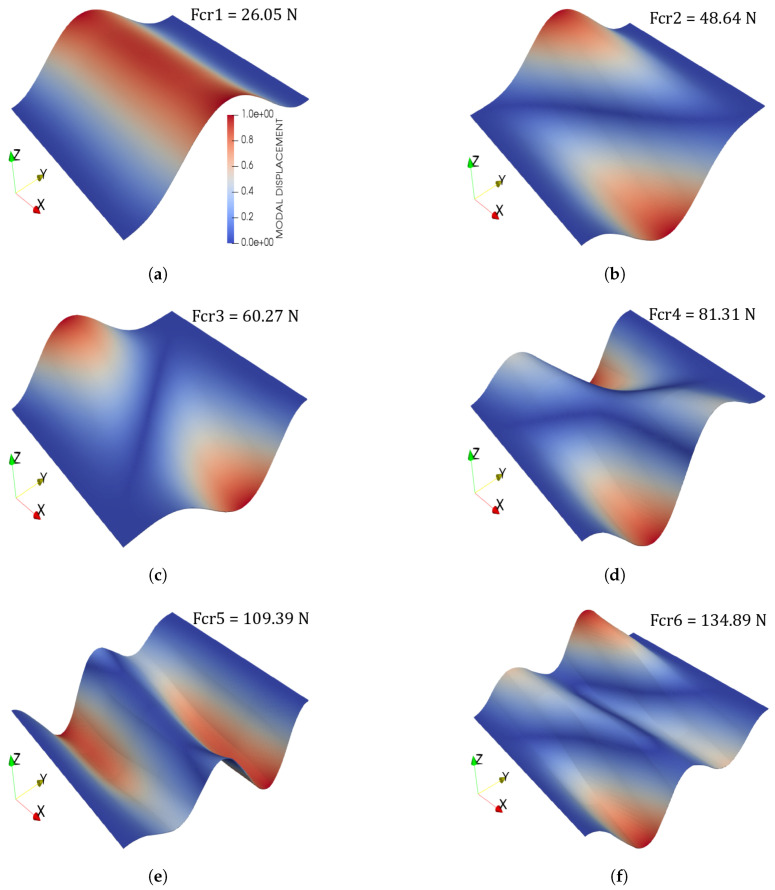
(**a**) First mode, (**b**) Second mode, (**c**) Third mode, (**d**) Fourth mode, (**e**) Fifth mode, (**f**) Sixth mode. Case 2 pristine buckling modes. A uniform fibre volume $V_{f}=0.60$ is considered and no fibre waviness. The colour bar in (**a**) applies for all figures in this panel.

Finally, buckling loads and modes of the two fibre paths are illustrated in Figure 11 and Figure 12. The following comments are made:

1. The proposed CUF model provides similar results to those obtained by commercial software.
2. The 4B4-4L16 mesh has been chosen because of its trade-off between accuracy and computational time.
3. Relevant differences in the buckling loads’ values appear when considering different rotation angle, that is, whether $\phi =0^{\circ }$ or $\phi =90^{\circ }$.

### 5.2. Single-Defect Multiscale Uncertainty

The second assessment considers the inclusion of fibre volume fraction stochastic fields in the numerical model to investigate their influence on the buckling loads. After performing $10^{3}$ Monte Carlo simulations, their outcomes are employed to compute some statistics, such as mean value and standard deviation. Additionally, these outcomes are used to build first- and second-order PCE, as explained in Section 4.1. The first two statistical moments, calculated through Monte Carlo analysis and first- and second-order PCE, are enlisted in Table 6 and Table 7. The standard deviation is expressed in terms of the Coefficient of Variation (COV), which is defined as the ratio between the standard deviation and the mean value.

**Figure 13:**
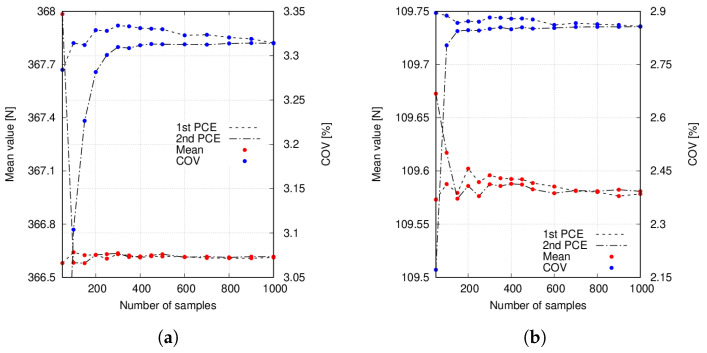
(**a**) Case 1 $F_{cr5}$, (**b**) Case 2 $F_{cr5}$. Mean value and COV convergence of the Case 1 and Case 2 buckling loads provided by the computed PCE for different amount of samples. Fibre volume fraction is treated as a Gaussian random variable with mean value $V_{f}=0.60$ and standard deviation $\sigma_{V_{f}}=0.05$ and no fibre waviness.

Additionally, the convergence of the mean value and COV concerning the number of samples used to build the PCE is reported in Figure 13.

**Figure 14:**
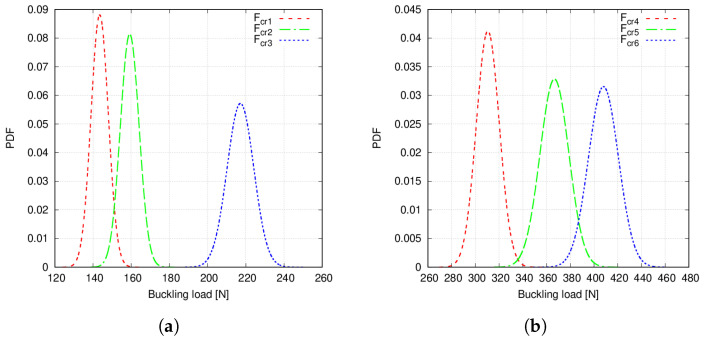
(**a**) Case 1 $F_{cr1}$, $F_{cr2}$ and $F_{cr3}$ PDFs, (**b**) Case 1 $F_{cr4}$, $F_{cr5}$ and $F_{cr6}$ PDFs. Fibre volume fraction is treated as a Gaussian random variable with mean value $V_{f}=0.60$ and standard deviation $\sigma_{V_{f}}=0.05$ and no fibre waviness.

**Figure 15:**
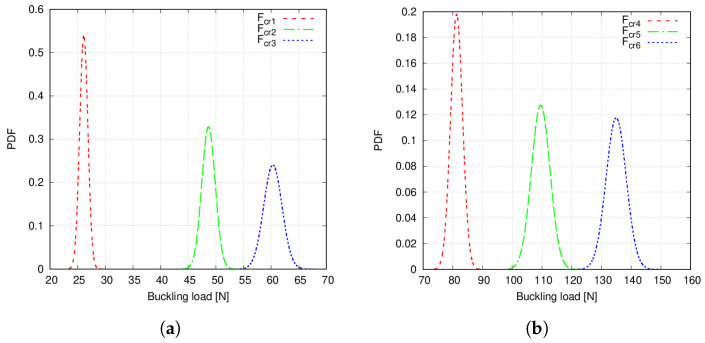
(**a**) Case 2 $F_{cr1}$, $F_{cr2}$ and $F_{cr3}$ PDFs, (**b**) Case 2 $F_{cr4}$, $F_{cr5}$ and $F_{cr6}$ PDFs. Fibre volume fraction is treated as a Gaussian random variable with mean value $V_{f}=0.60$ and standard deviation $\sigma_{V_{f}}=0.05$ and no fibre waviness.

Probability density functions (PDFs) are calculated from the Monte Carlo data and after carrying out $10^{4}$ emulations using the previous PCE. Note that with the term emulation, the authors refer to the surrogate model feeding and gathering results. These curves are represented in Figure 14 and Figure 15. For the sake of clearness, only the PDFs obtained with the second-order PCE emulation results are shown since Monte Carlo and PCE provide practically the same outcomes.

The computational time needed to perform the Monte Carlo analysis, obtain 300 samples to construct the PCE surrogate and emulate $10^{4}$ samples with PCE is enlisted in Table 8.

It is appreciated in Figure 14 that PDFs present overlapping tails, which is due to the fibre volume fraction variation. However, it is not clear whether the microscale uncertainty causes mode switching or not. For that purpose, correlation indices between the buckling load of the overlapping curves are calculated as follows:

(46) $$r_{j,k}=\frac{\sum_{i=1}^{Nsim}\left(F_{cr_{j}}^{i}-{\tilde{F}}_{cr_{j}}\right)\left(F_{cr_{k}}^{i}-{\tilde{F}}_{cr_{k}}\right)}{\sqrt{\sum_{i=1}^{Nsim}{\left(F_{cr_{j}}^{i}-{\tilde{F}}_{cr_{j}}\right)}^{2}}\sqrt{\sum_{i=1}^{Nsim}{\left(F_{cr_{k}}^{i}-{\tilde{F}}_{cr_{k}}\right)}^{2}}},$$

in which $F_{cr_{j}}^{i}$ is the *i*-th result for the *j*-th buckling load, ${\tilde{F}}_{cr_{j}}$ is the mean value of the *j*-th buckling load and $r_{j,k}$ represents the correlation between the *j*-th and *k*-th buckling load. Correlation results are enlisted in Table 9.

**Figure 16:**
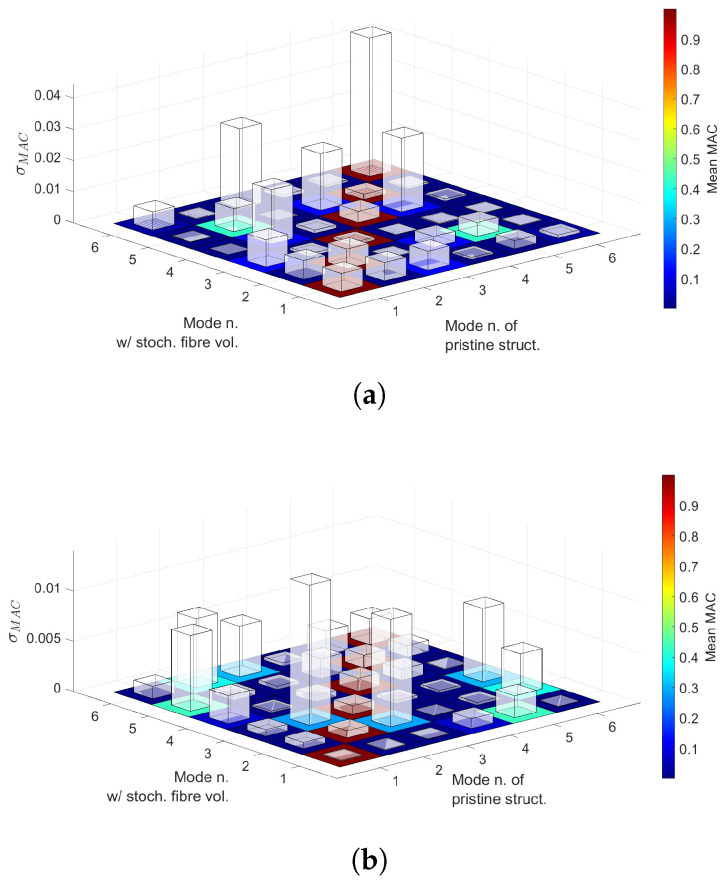
(**a**) Case 1 3D MAC matrix, (**b**) Case 2 3D MAC matrix. Floor indicates the mean value and bars the standard deviation of each component of the matrix. Fibre volume fraction is treated as a Gaussian random variable with mean value $V_{f}=0.60$ and standard deviation $\sigma_{V_{f}}=0.05$ and no fibre waviness.

Modal Assurance Criterion (MAC) matrix is used to foresee eventual mode switching and interactions between modes of the defected and the pristine structure. The matrix’s components are calculated as:

(47) $${\mathrm{MAC}}_{j,k}^{\left(i\right)}=\frac{|\phi_{i,j}^{T}\phi_{\mathrm{ref},k}{|}^{2}}{\left(\phi_{i,j}^{T}\phi_{i,j}\right)\left(\phi_{\mathrm{ref},k}^{T}\phi_{\mathrm{ref},k}\right)},$$

where $\phi_{i,j}$ is the *i*-th sample of the *j*-th eigenvector, $\phi_{\mathrm{ref},k}$ refers to the *k*-th eigenvector of the reference solution and ${\mathrm{MAC}}_{j,k}^{\left(i\right)}$ represents the *i*-th sample of the j,k component of the MAC matrix. Mean value and standard deviation of each component are calculated and represented in Figure 16.

The following comments are made:

1. Statistical moments provided by first- and second-order PCE are in good agreement with the Monte Carlo results, as seen in Table 6 and Table 7.
2. The mean value provided by the considered PCE converges when some 200 to 300 samples are employed to construct such surrogates. Conversely, COV needs additional samples.
3. The results in Table 8 suggest that PCE could be used to decrease computational times.
4. Overlapping tails appear in Case 1 buckling load PDFs, as seen in Figure 14. Conversely, Case 2 buckling load PDFs do not show such feature, as appreciated in Figure 15.
5. Correlation indices in Table 9 and MAC statistics in Figure 16 suggest that no mode switching occurs.

### 5.3. Double-Defect Multiscale Uncertainty

The third numerical assessment aims to show the capabilities of the current defect modelling approach. Herein, two kinds of uncertainty are included, namely fibre volume fraction and fibre misalignment. This implies that microscale and mesoscale defects are considered. The latter defects were already studied by the authors in [37,38]. The influence that the combination of defects has on the buckling load is addressed in this section. For this analysis, the fibre volume fraction keeps the same mean value and standard deviation. At the same time, misalignments have a null mean value and a standard deviation $\sigma_{\theta }=1.5^{\circ }$, in accordance with the data obtained by Sutcliffe et al. [46]. Additionally, taking advantage of the capabilities demonstrated in terms of convergence by PCE in the previous section (recall Figure 13), only 300 simulations are carried out to build regression models and compute the first two statistical moments, which are enlisted in Table 10 and Table 11.

**Figure 17:**
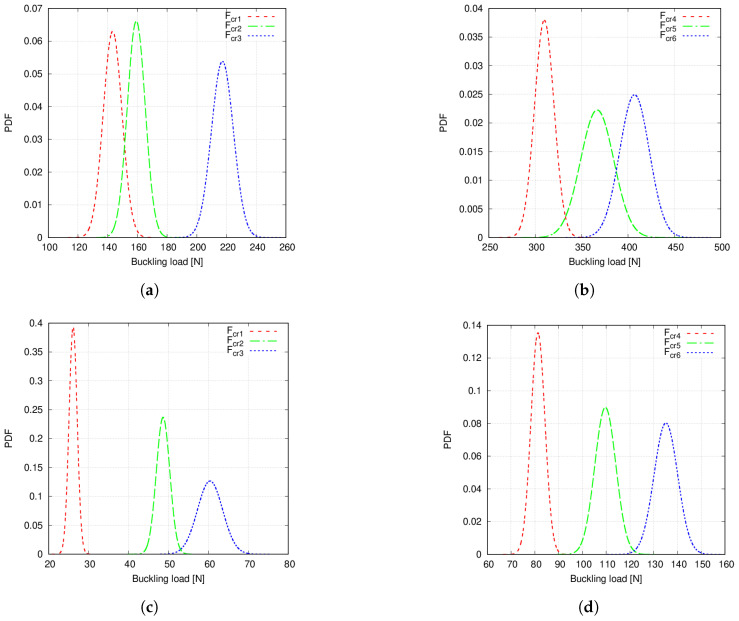
(**a**) Case 1 $F_{cr1}$, $F_{cr2}$ and $F_{cr3}$ PDFs, (**b**) Case 1 $F_{cr4}$, $F_{cr5}$ and $F_{cr6}$ PDFs. (**c**) Case 2 $F_{cr1}$, $F_{cr2}$ and $F_{cr3}$ PDFs. (**d**) Case 2 $F_{cr4}$, $F_{cr5}$ and $F_{cr6}$ PDFs. Fibre volume fraction is treated as a Gaussian random variable with mean value $V_{f}=0.60$ and standard deviation $\sigma_{V_{f}}=0.05$ whereas misalignments have a null mean and standard deviation $\sigma_{\theta }=1.5^{\circ }$.

Case 1 and 2 buckling load PDFs, considering the two defects, are represented in Figure 17. These PDFs were obtained using the second-order PCE surrogate and computing a total of $10^{4}$ emulations.

**Figure 18:**
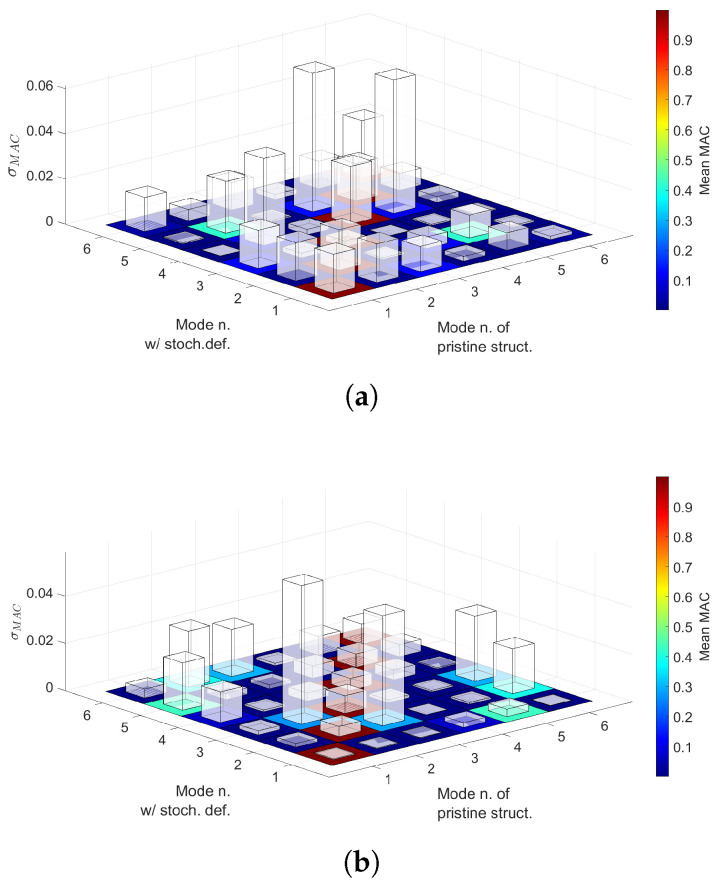
(**a**) Case 1 3D MAC matrix, (**b**) Case 2 3D MAC matrix. Floor indicates the mean value and bars the standard deviation of each component of the matrix. Fibre volume fraction is treated as a Gaussian random variable with mean value $V_{f}=0.60$ and standard deviation $\sigma_{V_{f}}=0.05$ whereas misalignments have a null mean and standard deviation $\sigma_{\theta }=1.5^{\circ }$.

As carried out in Section 5.2, mean value and standard deviation of each MAC matrix’s component are calculated and represented in Figure 18.

The following comments are made:

1. First- and second-order PCE provide similar results for both the mean and COV, as seen in Table 10 and Table 11. As expected, the addition of a second defect increases the COV of the buckling load distribution. Particularly, Case 2 shows a larger increment.
2. Case 1 and 2 buckling load PDFs are wider when two defects are accounted for, compared to the single-defect case. As a consequence, overlapping tails between PDFs appear now for both fibre paths. This can be appreciated in Figure 17.
3. MAC matrix’s statistics in Figure 18 suggest that no mode switching occurs, for both fibre paths, when two defects are accounted for.

## 6. Discussion

The first set of numerical assessments consists of the verification of the proposed methodology. It is observed in Table 3 and Table 4 that the present method provides results that are in agreement with those obtained by the commercial software Abaqus [51]. At first glance, it may seem that employing LW models in the characterisation of the buckling load of a VSC plate might not be helpful since, with the actual Abaqus shell model, a similar solution is achieved by using a fraction of the DOF. Nevertheless, this LW model approach is useful to include uncertainty at the micro and mesoscale level of the composite plate, which affects the internal stress state utilised to compute the geometric stiffness matrix to solve Equation (13). Therefore, a precise evaluation of such stresses is mandatory. The differences between Abaqus and CUF LW models stem from two main factors: (i) the modelling approach and (ii) the computation of the local fibre angle orientation. Regarding the former, an ESL approach is obtained with Abaqus since classic plate theories are employed in the definition of the FEs, whilst for the present LW model, a more accurate approach is used. Then, regarding the latter, in the Abaqus model, each ply element is assigned a specific fibre angle orientation based on the element’s centroid coordinates. Conversely, in the LW model, such value is computed at each integration point, leading to a more realistic modelling approach. LW models keep the local fibre orientation at the different plies, as opposed to the ESL approach, implemented in Abaqus, where a homogenisation of the layer orientations is carried out.

Some differences between Case 1 and Case 2 buckling loads arise and are commented herein. The clearest one is that Case 1 provides a larger critical load than Case 2. Indeed, it is one order of magnitude larger. This is due clearly to the fibre orientation and, particularly, to the rotation angle ϕ. That is, in Case 2, the fibre path is rotated 90 degrees with respect to the *x*-axis, which coincides with the loading direction. Thus, the load-carrying capacity of the plate diminishes. Further details regarding the stiffness and buckling load behaviour of symmetric and balanced VSCs can be found in the paper by Gürdal and Olmedo [52]. The buckling modes of the structure of both fibre angle orientations also present differences and are gathered in Figure 11 and Figure 12. It is appreciated that, for both cases, the first mode corresponds to a single-wave bending mode in the direction of the applied load. Then, the second mode presents a double-wave in the transverse in-plane direction and a single longitudinal undulation for both fibre paths. The third mode shows a triple undulation in the transverse in-plane direction in Case 1 (see Figure 11c), whereas a double one appears for Case 2 (see Figure 12c). More evident differences appear when higher modes are considered.

The second set of numerical results considers the spatial variation of the fibre volume fraction. Table 6 and Table 7 provide the mean value, and COV of the first six buckling loads for the two studied fibre paths. It is worth noting that similar values of COV are obtained. Additionally, Monte Carlo and PCE surrogates show a good agreement in the values of both statistical moments. Then, the convergence of the mean value and COV with regard to the number of samples used to build the PCE is reported in Figure 13. These results suggest that the computational cost of the uncertainty analysis can be strongly reduced by using such surrogates, as Table 8 shows. The number of terms for the first-order PCE is obtained by imposing r=40 and p=1, whereas r=40 and p=2 for second-order PCE. However, after computing the relative coefficients of such PCE, many of them were null. Hence, a reduction in the number of terms could be performed by employing sparse PCE, in which higher-order terms can be neglected. See [53] for further details.

Correlation indices reported in Table 9 have a value close to one. This indicates that when one of the confronted buckling loads increases, so does the other (e.g., $F_{cr1}$ and $F_{cr2}$ in $r_{1,2}$). This means that the spatial variation of fibre volume fraction affects the buckling loads in the same manner, that is, increasing or decreasing all of them. Then, concerning the mode variability, 3D MAC matrices in Figure 16 show mean values close to one in the main diagonal. Therefore, no mode switching occurs. However, out-of-the-diagonal components show non-zero values. For instance, in Figure 16a values close to 0.4 are appreciated in components MAC${}_{2,5}$ and MAC${}_{5,2}$, while in Figure 16b this occurs for MAC${}_{1,5}$, MAC${}_{2,3}$, MAC${}_{2,6}$, MAC${}_{3,6}$ and their transposed. This implies that the defective buckling modes show resemblances between the cited modes.

The third numerical assessment studied the presence of two spatially varying distributions of defects: fibre volume fraction and fibre misalignments. Second-order PCE was used to obtain the buckling load PDFs, illustrated in Figure 17. In these plots, some differences can be appreciated as compared to Figure 14 and Figure 15. As mentioned before, Case 1 PDFs considering both defects are wider than those of a single defect, which is due to the higher COV reported in Table 10 and Table 11. Again, overlapping PDF tails are still present and even more exacerbated since, in Figure 17b, upper and lower tails of the fourth and sixth critical loads slightly overlap around 350 N. Similarly, Case 2 PDFs are wider. Indeed, in this case, overlapping tails appear between the second and third loads and fourth, fifth and sixth loads, which did not occur in the precedent results.

Three dimensional MAC matrices, provided in Figure 18, inform that no swapping occurs between buckling modes. For both fibre patterns, main diagonal components have a mean value close to one, whilst some of the remaining components present values between 0.40 and 0.50. This implies that the defective modes have resemblances of the pristine ones. Concerning the standard deviation of the MAC components, Case 1 presents similar values to those obtained priorly, whilst, for Case 2, such standard deviation increased, which is in agreement with the behaviour of Case 2 COV of the buckling loads.

Finally, concerning the uncertainty results, additional information can be inferred. For instance, for the two analysed fibre orientations, a COV between 2.5% and 3.5% variability in the buckling loads was obtained for the single-defect uncertainty. Then, the double-defect uncertainty results provided a COV between 3.4% and 5.2%. This confirms that, for the studied cases, the consideration of multiple uncertain defects leads to broader stochastic structural responses. In this manner, in a 3σ reliability analysis, the buckling loads may vary up to 11.5% and 15% when microscale uncertainty and micro and mesoscale uncertainty are considered, respectively.

## 7. Conclusions

In this study, a 1D modelling strategy based on the Carrera Unified Formulation (CUF) framework has been proposed for the linearised buckling analysis of VSC plates. The LW CUF model of a four-layered cantilevered plate has been verified against commercial software Abaqus. The LW capabilities allow us to consider the local fibre orientation of each ply independently, which is helpful when considering defects.

The inclusion of uncertain defects was achieved by means of stochastic fields, thus yielding a non-intrusive technique of considering defects. In this manner, no additional degrees of freedom (DOF) are required, and the computational cost is kept invariable.

The presented methodology has been found to provide satisfactory results when dealing with multiscale uncertain defects for the buckling analysis of VSC plates. Moreover, this procedure will permit to conduct more complex structural problems involving such defects. Future work aims to investigate the macro and micro stress state of VSC laminates. Unfortunately, and to the authors’ concern, no experimental evidence of the structural response variability stemming from the defects herein considered has been found. Nevertheless, recent numerical works (see Dodwell et al. [54] and van der Broek et al. [55]) have addressed the influence of misalignments in the buckling and post-buckling regime, providing COV that are in agreement with the ones obtained in this manuscript.

Finally, the usage of PCE as regression metamodels for the buckling loads has been found to be an accurate tool, although it presents some drawbacks. Among them, the inclusion of different random fields to characterise diverse uncertainty defects increases the amount of polynomial basis that conform the PCE, which may lead to the so-called curse of dimensionality as discussed in Section 4.1. Therefore, additional surrogate models or techniques might be applied to circumvent that issue.

## Figures and Tables

**Table 1:** Geometrical dimensions of the laminated plate.

*a* [m]	*b* [m]	Ply Thickness [mm]
0.254	0.254	0.127

**Table 2:** Elastic properties of the constituents of the composite material and the homogenised material properties for a fibre volume fraction $V_{f}=0.60$.

Constituent	$E_{11}$ [GPa]	$E_{22}$ [GPa]	$G_{12}$ [GPa]	$G_{23}$ [GPa]	$\nu_{12}$ [-]	$\nu_{23}$ [-]
Fibre	235.0	14.0	28.0	5.60	0.20	0.25
Matrix	4.80	4.80	1.79	1.79	0.34	0.34
Homogenised $V_{f}$ = 0.60	143.17	9.64	6.09	3.12	0.252	0.349

**Table 3:** Convergence of the buckling load for the Case 1 pristine structure. A uniform fibre volume $V_{f}=0.60$ is considered and no fibre waviness.

Model	Mesh	DOF	$F_{\mathrm{cr}1}$ [N]	$F_{\mathrm{cr}2}$ [N]	$F_{\mathrm{cr}3}$ [N]	$F_{\mathrm{cr}4}$ [N]	$F_{\mathrm{cr}5}$ [N]	$F_{\mathrm{cr}6}$ [N]
Abaqus	11 × 11	726	154.82	175.73	240.64	355.42	397.41	481.44
	14 × 14	1176	146.08	166.74	229.19	332.39	373.74	452.42
	26 × 26	4056	141.92	161.34	220.12	310.66	349.98	422.85
	35 × 35	7350	134.29	151.70	215.58	287.20	321.01	412.78
	52 × 52	16,224	133.80	151.11	214.76	285.49	318.71	410.53
Cubic	2B4-2L16	2184	163.98	186.71	255.72	391.69	440.89	521.07
	4B4-4L16	7098	143.59	159.08	216.77	310.22	366.45	407.53
	6B4-6L16	14,820	142.01	156.24	211.10	294.06	349.56	390.31
	8B4-8L16	25,350	141.64	155.57	209.82	290.89	345.89	386.11
Quadratic	4B3-4L9	2430	209.87	236.55	311.29	436.55	501.59	570.80
	6B3-6L9	4914	168.20	187.70	251.96	367.75	414.15	477.14
	8B3-8L9	8262	155.93	172.94	232.71	333.21	384.60	436.97
	10B3-10L9	12,474	150.49	166.32	223.96	316.89	370.03	417.18
	12B3-12L9	17,550	145.85	160.64	216.41	302.76	356.92	400.51

**Table 4:** Convergence of the buckling load for the Case 2 pristine structure. A uniform fibre volume $V_{f}=0.60$ is considered and no fibre waviness.

Model	Mesh	DOF	$F_{\mathrm{cr}1}$ [N]	$F_{\mathrm{cr}2}$ [N]	$F_{\mathrm{cr}3}$ [N]	$F_{\mathrm{cr}4}$ [N]	$F_{\mathrm{cr}5}$ [N]	$F_{\mathrm{cr}6}$ [N]
Abaqus	11 × 11	726	26.49	51.56	64.29	88.30	137.57	168.73
	14 × 14	1176	25.75	49.55	61.27	83.54	121.31	149.29
	26 × 26	4056	24.99	47.35	58.64	78.90	107.40	132.03
	35 × 35	7350	24.71	47.02	57.67	78.09	104.31	129.26
	52 × 52	16,224	24.67	46.75	57.55	77.59	103.26	127.54
Cubic	2B4-2L16	2184	27.90	59.06	91.21	141.54	322.77	387.92
	4B4-4L16	7098	26.05	48.64	60.27	81.31	109.39	134.89
	6B4-6L16	14,820	25.42	46.97	58.29	77.04	108.59	128.68
	8B4-8L16	25,350	25.21	46.49	57.75	76.33	105.89	124.91
Quadratic	4B3-4L9	2430	34.83	69.15	89.79	137.56	323.65	391.87
	6B3-6L9	4914	29.65	56.91	72.49	101.51	154.18	196.38
	8B3-8L9	8262	27.77	52.52	65.55	89.78	134.59	163.93
	10B3-10L9	12,474	26.84	50.33	62.49	84.59	123.75	148.71
	12B3-12L9	17,550	26.33	49.13	60.92	81.87	117.82	140.63

**Table 5:** Dimensionless CPU time for the different meshes employed with the present LW approach.

Model	Mesh	DOF	$t/t_{\mathrm{ch}}$ [-]
Cubic	2B4-2L16	2184	0.30
	4B4-4L16	7098	1.00
	6B4-6L16	14,820	2.47
	8B4-8L16	25,350	4.23
Quadratic	4B3-4L9	2430	0.01
	6B3-6L9	4914	0.22
	8B3-8L9	8262	0.37
	10B3-10L9	12,474	0.55
	12B3-12L9	17,550	0.79

**Table 6:** Case 1 critical buckling loads mean value and standard deviation calculated by means of pure Monte Carlo and first- and second-order PCE.

Buckling	Deterministic	Monte Carlo	1st Order PCE	2nd Order PCE	Monte Carlo	1st Order PCE	2nd Order PCE
Load	Value [N]	Mean [N]	Mean [N]	Mean [N]	COV [%]	COV [%]	COV [%]
$F_{cr_{1}}$	143.59	143.48	143.48	143.49	3.14	3.16	3.16
$F_{cr_{2}}$	159.07	159.21	159.21	159.21	3.07	3.08	3.09
$F_{cr_{3}}$	216.77	217.16	217.16	217.16	3.20	3.22	3.21
$F_{cr_{4}}$	310.21	310.47	310.46	310.48	3.11	3.12	3.12
$F_{cr_{5}}$	366.45	366.61	366.61	366.62	3.27	3.31	3.31
$F_{cr_{6}}$	407.53	407.91	407.91	407.93	3.24	3.11	3.10

**Table 7:** Case 2 critical buckling loads mean value and standard deviation calculated by means of pure Monte Carlo and first- and second-order PCE.

Buckling	Deterministic	Monte Carlo	1st Order PCE	2nd Order PCE	Monte Carlo	1st Order PCE	2nd Order PCE
Load	Value [N]	Mean [N]	Mean [N]	Mean [N]	COV [%]	COV [%]	COV [%]
$F_{cr_{1}}$	26.05	26.10	26.10	26.10	2.81	2.84	2.84
$F_{cr_{2}}$	48.64	48.66	48.66	48.66	2.49	2.49	2.50
$F_{cr_{3}}$	60.27	60.30	60.29	60.30	2.75	2.76	2.75
$F_{cr_{4}}$	81.31	81.29	81.29	81.29	2.48	2.48	2.48
$F_{cr_{5}}$	109.3	109.58	109.58	109.58	2.85	2.86	2.86
$F_{cr_{6}}$	134.89	134.96	134.96	134.96	2.52	2.51	2.51

**Table 8:** Computational time needed to: perform a Monte Carlo analysis consisting of $10^{3}$ samples; obtain 300 samples to construct the PCE surrogate, and emulate $10^{4}$ samples using PCE.

Monte Carlo [hours]	PCE Build-Up [hours]	PCE Usage [s]
71	21	3

**Table 9:** Correlation indices between Case 1 buckling loads.

$r_{1,2}$	$r_{4,5}$	$r_{5,6}$
0.998	0.912	0.988

**Table 10:** Case 1 critical buckling loads mean value and COV calculated by means of first- and second-order PCE considering spatially varying fibre volume fraction and fibre misalignments.

Buckling	Deterministic	1st Order PCE	2nd Order PCE	1st Order PCE	2nd Order PCE
Load	Load [N]	Mean [N]	Mean [N]	COV [%]	COV [%]
$F_{cr_{1}}$	143.59	143.30	143.34	4.52	4.42
$F_{cr_{2}}$	159.07	159.29	159.26	3.91	3.79
$F_{cr_{3}}$	216.77	217.22	217.23	3.61	3.41
$F_{cr_{4}}$	310.21	309.82	309.76	3.59	3.39
$F_{cr_{5}}$	366.45	366.75	366.76	5.02	4.88
$F_{cr_{6}}$	407.53	407.09	406.99	4.05	3.93

**Table 11:** Case 2 critical buckling loads mean value and COV calculated by means of first- and second-order PCE considering spatially varying fibre volume fraction and fibre misalignments.

Buckling	Deterministic	1st Order PCE	2nd Order PCE	1st Order PCE	2nd Order PCE
Load	Load [N]	Mean [N]	Mean [N]	COV [%]	COV [%]
$F_{cr_{1}}$	26.05	26.13	26.14	3.96	3.88
$F_{cr_{2}}$	48.64	48.65	48.65	3.57	3.46
$F_{cr_{3}}$	60.27	60.42	60.41	5.32	5.22
$F_{cr_{4}}$	81.31	81.32	81.32	3.72	3.63
$F_{cr_{5}}$	109.30	109.67	109.66	4.08	4.05
$F_{cr_{6}}$	134.89	135.11	135.11	3.75	3.68

## Data Availability

The data presented in this study are available on request from the corresponding author.
